# From Salivary Dysfunction to Prosthetic Challenges in Xerostomia and Denture Retention with Oral Gels

**DOI:** 10.3390/ma18133141

**Published:** 2025-07-02

**Authors:** Dawid Łysik, Joanna Niewęgłowska, Joanna Mystkowska

**Affiliations:** Institute of Biomedical Engineering, Bialystok University of Technology, Wiejska 45C, 15-351 Bialystok, Poland; d.lysik@pb.edu.pl (D.Ł.); joanna.nieweglowska@sd.pb.edu.pl (J.N.)

**Keywords:** saliva, dental, drug safety, oncology, health care, cancer, life expectancy

## Abstract

Xerostomia significantly compromises oral comfort, mucosal integrity, and denture retention. While topical therapies such as oral gels are commonly used to manage symptoms, their effectiveness remains limited due to an inability to replicate the complex biochemical and mechanical functions of natural saliva. This review explores the pathophysiology of salivary dysfunction, the structural and functional roles of mucins, and the tribological and rheological demands of the oral environment—particularly in denture wearers. Emphasis is placed on the interplay between mucosal surfaces, salivary films, and prosthetic biomaterials, as well as the importance of mucoadhesion and aqueous boundary lubrication. A rheological comparison of commercially available oral gels and whole human saliva (WHS) reveals that gels are significantly more viscous and elastic, yet fail to mimic the dynamic responsiveness of saliva. Current formulations lack functional standardization and labeling, limiting clinical guidance. The study proposes design principles for next-generation gels that incorporate amphiphilic, biomimetic components and measurable performance benchmarks.

## 1. Introduction

Saliva is a complex biological fluid composed of approximately 99% water and 1% proteins, including enzymes (e.g., amylase, lysozyme), mucins, immunoglobulins, and growth factors, along with trace amounts of ions and electrolytes. It acts as a natural lubricant that facilitates chewing, swallowing, speaking, and the initiation of digestion, while its antimicrobial and mineral-rich composition is crucial for the maintenance and remineralization of dental enamel [1,2]. Saliva also contains signaling molecules such as histatins that contribute to the repair and regeneration of the oral and esophageal mucosa [3,4,5]. This is particularly important in the oral cavity, where wounds—resulting from extractions, biting, or chronic irritation from prosthetics—heal faster and with fewer complications than comparable injuries elsewhere in the body [6,7]. Saliva supports this process by maintaining a moist environment conducive to inflammatory cell activity and by providing glycosylated proteins, carbohydrates, enzymes, growth factors, and antimicrobial peptides that promote efficient tissue repair [8]. Furthermore, the molecular composition of saliva reflects the physiological and pathological status of the body, making it a valuable noninvasive source of biomarkers for disease detection [8,9].

However, the importance of saliva becomes most evident when its production is reduced or lost. Xerostomia, commonly known as dry mouth, is the subjective sensation of oral dryness reported by patients and does not always correlate with measurable changes in saliva output. In contrast, hyposalivation is an objective, clinically defined decrease in salivary flow—typically below 0.1 mL/min—which carries a wide range of health consequences. These include impaired taste perception, difficulty swallowing and speaking, cracked lips, oral mucosal irritation, tongue papillae loss, enamel erosion, increased dental caries, oral infections, halitosis, gingivitis, mucosal ulcers, and even malnutrition. Together, these complications highlight the essential role of saliva not only in oral function but also in maintaining systemic health and quality of life [10,11].

The prevalence of xerostomia varies widely, affecting between 10% and 25% of the general population, with some studies reporting rates as high as 43.6% in adults attending primary care [12]. Older adults are particularly vulnerable: one study reported an average age of 59 among xerostomia patients, compared to 53 among those without symptoms. Women are also disproportionately affected, being 1.64 times more likely to report dry mouth than men [12].

Importantly, poor oral health—including salivary dysfunction—is increasingly recognized as a contributing factor to frailty in older adults, which refers to a state of increased vulnerability due to age-related decline in physiological reserves. A recent systematic review of 39 studies involving individuals over 60 years identified 12 oral health indicators associated with frailty [13]. These were grouped into four domains: (1) deterioration of oral health status (e.g., tooth loss), (2) decline in oral motor skills (e.g., masticatory efficiency, occlusal force), (3) disorders of chewing, swallowing, and saliva, and (4) oral pain. Among these, tooth loss (especially having few remaining teeth) and chewing difficulties were most consistently linked to frailty. These findings suggest that salivary dysfunction may not only impair daily oral function but may also contribute to broader physical, cognitive, and nutritional decline—further underscoring the clinical importance of early recognition and management of xerostomia and hyposalivation in aging populations.

Beyond its evident consequences for oral health, xerostomia presents unique challenges for individuals who rely on prosthetic restorations, particularly complete dentures. Saliva plays a pivotal role in denture retention and stability by serving as a natural lubricant and ensuring adequate adhesion between the denture and the oral mucosa [14]. The lubricating properties of saliva are essential for minimizing friction and ensuring smooth movement of the denture against the underlying tissues. In the absence of sufficient saliva, denture wearers frequently encounter a range of issues primarily stemming from the lack of this natural lubrication.

The absence of adequate lubrication can lead to increased friction between the denture and the underlying tissues, resulting in pain, abrasions, and even ulcerations. The study by Al-Dwairi and Lynch [15] further supports this, indicating that 73.5% of xerostomic participants reported feeling soreness, compared to only 15% among participants without xerostomia. The constant rubbing and irritation can significantly impair the wearer’s comfort and ability to function normally. Moreover, saliva aids in securing the denture in place by creating a “fluid seal” and enhancing adhesive forces. In cases of xerostomia, the denture may shift, dislodge, or even cause difficulties with speech and eating due to compromised retention [16]. Importantly, several studies [17,18,19] have shown that not only reduced salivary flow but also alterations in its physicochemical properties—such as lower viscosity, diminished surface tension, or impaired wettability—can significantly weaken the adhesive and cohesive mechanisms necessary for stable prosthesis function.

Although the literature describes a variety of advanced approaches to restoring salivary function—including pharmacological therapies [20,21], cell- and tissue-based regenerative strategies [22], cell transplantation [23,24,25], and gene therapy [26]—these remain largely experimental. In routine clinical practice, symptomatic management of xerostomia in denture wearers relies predominantly on topical methods. These include frequent water sipping, the use of saliva substitute sprays, and the application of oral moisturizing gels [27]—the latter emerging as particularly promising.

Oral gels containing moisturizing and coating substances may offer a promising option for alleviating dry mouth symptoms and improving denture comfort. However, it is important to remember that the surfaces of prosthetic components differ significantly from natural oral tissues in terms of their physicochemical properties [14,28].

Materials used in prosthetics, such as acrylic polymers or metal alloys, have different wettability, which is crucial for the effectiveness of lubrication. Furthermore, the abiotic surfaces of dentures exhibit distinct chemical properties compared to biological tissues, influencing the adsorption of salivary components and interactions with the oral microflora [29,30].

These differences can lead to faster biofilm formation on denture surfaces, increasing the risk of infections and mucosal irritation [31,32]. Therefore, it is crucial that the moisturizing and lubricating agents used are appropriately selected for the specific prosthetic materials and consider the individual needs of the patients.

As part of this narrative review, literature was searched primarily via PubMed, focusing on xerostomia, oral lubrication, tribology, rheology, and oral gels. Studies were selected based on conceptual relevance rather than predefined inclusion/exclusion criteria.

The following sections examine the physiology and dysfunction of salivary secretion, the structural and molecular properties of saliva, and the role of mucins in lubrication and mucoadhesion. This forms the basis for evaluating the rheological and tribological performance of oral gels and their potential to restore function and comfort in patients with dry mouth and denture-related challenges.

## 2. Physiology and Dysregulation of Salivary Secretion

### 2.1. Salivary Gland Function and Saliva Composition

Over 90% of the roughly 1.5 L of saliva produced daily in humans comes from three major pairs of salivary glands: the parotid, submandibular, and sublingual glands [33]. The parotid gland is the largest and is primarily responsible for stimulating saliva secretion. It produces a watery, enzyme-rich serous fluid that contains amylase. The submandibular gland produces most of the resting, or basal, saliva—a mix of serous and mucous secretions. The sublingual gland produces only mucous saliva, which is rich in salivary mucins. The remaining 10% of saliva comes from hundreds of minor salivary glands scattered throughout the oral cavity [34].

The function of the salivary glands in producing and secreting saliva depends on tightly coordinated interactions between multiple specialized cell types. These cells work together to generate a primary fluid—resembling plasma—which is subsequently modified into final saliva that is hypotonic, nearly neutral in pH, mucous-rich, viscous, and supersaturated with calcium (Ca^2+^) and phosphate ions. This complex secretory physiology is driven by ionic gradients that facilitate water transport, selective reabsorption of ions, and secretion of mucins for lubrication, along with calcium- and phosphate-binding proteins that help maintain tooth mineralization and oral supersaturation [33,35].

Saliva contains hundreds of proteins and peptides, many of which have distinct biological functions. Recent advances in single-cell transcriptomics have enabled precise mapping of these proteins to their cell of origin. Acinar cells are primarily responsible for synthesizing and secreting digestive enzymes, antimicrobial proteins, and immunomodulatory factors such as amylase, lysozyme, lactoferrin, mucins, histatins, cystatins, statherin, and secretory IgA. Ductal cells further modify the ionic composition of saliva and produce specific proteins, including carbonic anhydrases and kallikreins. Myoepithelial cells, located around both acini and ducts, provide structural support and assist in the expulsion of saliva by contracting in response to neural stimuli [36].

### 2.2. Etiology of Salivary Gland Dysfunction

A wide range of systemic diseases and local pathologies can compromise glandular tissue integrity and lead to salivary hypofunction, often reflected in both decreased volume and altered composition of saliva. Iatrogenic causes, such as radiation therapy for head and neck cancers, are especially damaging and may result in permanent gland dysfunction [37]. Currently, no curative treatments exist for radiation-induced damage, and available therapies remain predominantly palliative [33,38].

Depending on the underlying etiology, salivary gland disorders may be developmental, autoimmune, mechanical, or oncogenic in nature, and their clinical course may be temporary, chronic, or irreversible. One of the most commonly reported symptoms is xerostomia, the subjective sensation of dry mouth, which can arise from various factors that impair glandular function. These include infections, duct obstruction, systemic and autoimmune diseases, tumors, and cancer treatments such as radiotherapy and chemotherapy. In many cases, xerostomia coexists with hyposalivation—an objectively measurable reduction in salivary flow—further exacerbating the impact on oral health and quality of life [33].

In younger individuals, xerostomia is relatively uncommon and, when present, is most often associated with the use of medications. In people around the age of 30, the prevalence of xerostomia has been reported at approximately 10%, but this rate was found to be 22 times higher among those taking antidepressants [39]. Increased incidence of dry mouth has also been observed in individuals using iron supplements and opioid analgesics. Given that the nervous system plays a key role in regulating salivary secretion, it is not surprising that psychological and neurological factors such as stress, anxiety, or depression can contribute to the development of xerostomia [40]. Neural stimulation is essential for proper glandular secretion, and damage to the central nervous system—such as from encephalitis or brain tumors—can impair this function [41,42]. Other contributing factors include tobacco use [43] and a wide range of medications, including antihistamines, antihypertensives, antidepressants, antiepileptics, anxiolytics, anticholinergics, and antimuscarinic agents [44]. Sympathomimetic drugs may also alter the volume and consistency of saliva, often promoting the production of thicker, more mucous-dominant secretions [40,45]. Infections may cause salivary gland inflammation, further impairing function [46,47].

Mechanical injury leading to obstruction of the salivary ducts may result in a temporary loss of glandular function, which typically resolves after the blockage is removed [48,49]. However, if left untreated, obstruction can lead to fibrosis and permanent glandular dysfunction. Xerostomia is significantly more common in older adults, particularly those living in long-term care facilities, where prevalence rates have been reported as high as 60%. While aging itself is not directly associated with reduced salivary output, studies have shown that decreased function of the parotid glands under stimulation is more closely related to chronic disease burden and polypharmacy [33].

### 2.3. Radiation-Induced Salivary Dysfunction

Permanent loss of salivary function is more likely in cases involving congenital disorders such as gland agenesis, autoimmune diseases like Sjögren’s syndrome or graft-versus-host disease, and systemic conditions including diabetes and cystic fibrosis [27]. In addition, radiation therapy for head and neck cancers and the presence of salivary gland tumors can lead to irreversible damage [50]. Globally, approximately 59,660 new cases of oral cavity and pharynx cancers are diagnosed each year [51]. More than half of these tumors are benign, with the majority originating in the parotid glands. Among malignant forms, mucoepidermoid carcinoma, adenocarcinoma, and adenoid cystic carcinoma are the most common [52].

Surgical removal remains the standard treatment for these tumors, often followed by radiation therapy to control locoregional spread [50]. Given the high risk of recurrence, treatment of head and neck cancers often involves relatively high cumulative doses of ionizing radiation, typically between 66 and 74 Gy [50]. Acute hyposalivation, or rapid loss of saliva production, often begins within the first week of treatment, resulting in up to a 60% reduction in output. In nearly all patients, mucositis develops by the third week. Chronic hyposalivation may last for weeks or months and, in many cases, becomes permanent. This leads to a cascade of complications that severely impact quality of life and often cause psychological distress. Radiation-induced salivary gland dysfunction remains a major clinical challenge [38,53,54].

The extent of glandular damage depends on the total radiation dose received. Studies have estimated that up to 40% of salivary function can be preserved 1 year after treatment if the cumulative dose to the parotid gland does not exceed 50 Gy. Full recovery is possible when the dose remains below 25 to 30 Gy [55]. Animal studies have consistently shown that acute hyposalivation results from a combination of factors, including DNA damage [56], death of acinar cells [57,58], elevated production of reactive oxygen species (ROS) [59], and disrupted calcium signaling [60]. These biological insults collectively impair the secretory machinery of the gland, leading to rapid and often irreversible dysfunction.

## 3. Functional Components of Saliva and the Basis for Oral Gels

### 3.1. Salivary Components and Their Functional Roles

Efforts to restore salivary secretion or replace its lost function have been widely explored. Currently, oral lubricants, saliva substitutes, and stimulants remain the mainstay of palliative care for patients with xerostomia. Lubricants such as rinses, gels, and toothpastes are primarily aimed at protecting the oral mucosa, while saliva substitutes attempt to replicate the broader physiological roles of natural saliva beyond hydration [27].

However, clinical evidence supporting the effectiveness of these topical therapies is limited. Recent studies [61] show that while most oral gels offer only short-term relief, only a small fraction demonstrated superior performance compared to water. Notably, improved outcomes were seen with formulations containing agents such as porcine gastric mucin, carrageenan, carboxymethylcellulose, xanthan gum, or carbomer, which enhance moisture retention through their bioadhesive and gel-forming properties.

An earlier systematic review [62] had already suggested that topical therapies for xerostomia are generally of limited effectiveness, concluding that no such treatment consistently alleviates dry mouth symptoms. Some evidence suggests that oxygenated glycerol triester spray [63] may be more effective than electrolyte-based sprays, and chewing gums may stimulate secretion in patients with residual gland function, though no significant advantage over saliva substitutes has been shown. Integrated oral care devices show potential but require further validation.

Once natural saliva is secreted, modified, and delivered to the oral cavity, it plays a central role in lubrication [64]. Adequate hydration of the oral environment enables effective mastication, bolus formation, and swallowing, while also protecting mineralized tooth surfaces and preserving the integrity of the oral epithelium. This lubricating function is largely attributed to the heavy glycosylation of many salivary proteins, particularly mucins.

The salivary proteome comprises thousands of proteins and peptides [65,66]. Major functional protein families include mucins, proline-rich proteins (both acidic and basic), salivary amylase, agglutinins, secretory immunoglobulins, cystatins, histatins, and statherins. The rich glycan landscape of the oral cavity is predominantly derived from mucins, which are the most heavily glycosylated salivary proteins. The primary mucins in human saliva are MUC5B—a high-molecular-weight gel-forming mucin—and MUC7, a soluble, low-molecular-weight mucin. Other mucins such as MUC19, MUC1, and MUC4 are also present [67].

### 3.2. Mucins: Structure, Glycosylation, and Role in Oral Lubrication

Mucins are highly glycosylated, with O-linked glycans—attached to serine and threonine residues—accounting for up to 80% of their dry molecular mass, giving them a bottlebrush-like structure. They also contain N-glycans and various sialoglycans, including terminal sialic acid, sulfate groups, and O-acetyl modifications. These confer a net negative charge and strong water-binding capacity, allowing mucins to retain moisture and give saliva its characteristic viscoelastic and rheological properties [68].

Beyond hydration, mucins and other salivary proteins coat the oral tissues to form a thin, protective film known as the acquired pellicle. This barrier plays a key role in preventing desiccation, maintaining mineral balance, regulating bacterial colonization, and protecting oral tissues from dietary and bacterial acids [69].

Studies in patients with Sjögren’s syndrome have shown that although mucin concentrations may remain similar to healthy individuals, the glycoprotein structure is often altered. Specifically, there is a decrease in O-glycan content and sialic acid residues [70]. This deglycosylation reduces mucin viscosity and its hydrating properties, compromising its biological function. Similarly, cryo-scanning electron microscopy in patients who received head and neck radiation therapy revealed thinner mucin fibers that form a weaker, more porous network compared to the dense, robust structure seen in healthy saliva. The loss of negatively charged glycans appears to impair mucin water retention, contributing to the sensation of oral dryness and decreased mucosal hydration [71,72].

**Figure 1:**
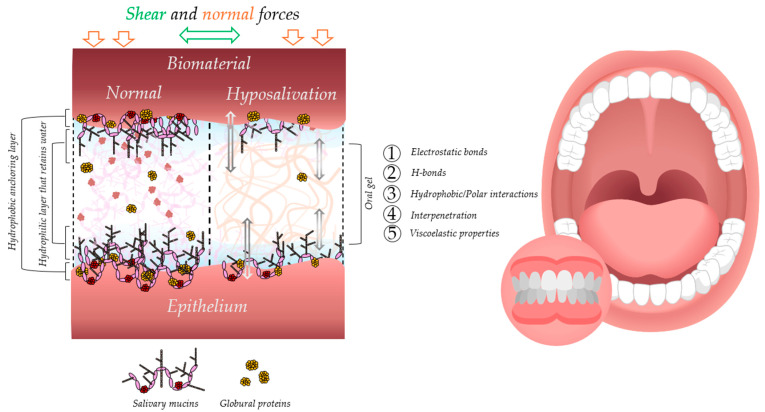
Schematic representation of the interfacial interactions between an oral gel, salivary components, mucosal tissue, and prosthetic biomaterials under normal and xerostomic conditions. In healthy saliva, a two-layered adsorbed film forms, with a hydrophobic anchoring zone (composed of mucin core regions and globular proteins) and a hydrated glycosylated zone responsible for water retention and boundary lubrication. In xerostomia, reduced salivary volume and altered mucin glycosylation compromise film integrity, hydration, and adhesion. The applied gel should interpenetrate the residual film, mimic mucin amphiphilicity and electrostatic interactions, and restore hydration and lubricity on both biotic and abiotic surfaces.

The critical role of mucins in biological lubrication has driven efforts to replicate their structure and function. Soluble, gel-forming mucins are composed of two key elements: a protein backbone and densely grafted glycans (Figure 1). These structural features determine the polymer’s charge, stiffness, molecular weight, and pore size—properties that govern the viscoelasticity, lubricity, and barrier function of mucin hydrogels. Glycan composition and density further modulate interactions with microorganisms and immune cells.

Numerous studies have attempted to dissect how mucin conformation and the arrangement and density of glycans influence specific biological functions, including microbial adhesion, immune modulation, and barrier integrity [73,74,75,76]. Although full synthetic replication of native mucins remains unattainable due to their immense structural complexity—particularly the diversity and specificity of glycosylation—deconstructing mucins into functional units enables systematic design of mucin mimetics [77]. In this context, both the protein backbone and glycan components appear essential for achieving effective lubrication [78].

To replicate mucin-like behavior, various non-peptidic polymer backbones have been explored for their ability to reproduce the mechanical and barrier-forming properties of native mucins. Materials such as carboxymethylcellulose [79], hyaluronan [80], guar gum [81,82], and xanthan gum [83,84,85] have demonstrated partial success in mimicking mucin’s viscoelasticity and lubricity. However, nonglycosylated hydrogels fall short in replicating the full spectrum of mucin’s biological activities—especially those mediated by glycan structures—though they may still be suitable for creating a mechanically robust barrier.

Understanding the rheological and lubricating properties of saliva, as well as the impact of mucin glycosylation changes, is essential for the development of effective saliva substitutes. These findings provide a scientific rationale for designing oral gels and related products that can mimic the protective and hydrating functions of natural mucins in xerostomia management.

## 4. Adsorption of Salivary Pellicle

The inevitable consequence of the presence of saliva in the oral cavity is the adsorption of its components (over 1500 known proteins) onto surfaces such as teeth and dental biomaterials [86]. Salivary pellicle (SP) acts as a protective coating for oral surfaces against wear and aids in speech and chewing [87]. SP can reduce the rate of enamel demineralization and inhibit the “surface-induced” precipitation of calcium-rich substances, leading to excessive mineralization of tooth surfaces [88]. Furthermore, SP controls cariogenic biofilm formation on teeth and dental biomaterials and may influence the healing of periodontal wounds [69,89].

The aspects related to biomaterials are fundamental because they exhibit different surface properties than natural ones [90,91]. Over 45% of patients undergoing orthodontic treatment developed caries due to biofilm formation on orthodontic materials [92]. Similarly, studies have shown that removable partial dentures in the oral cavity increase biofilm formation and, consequently, the occurrence of caries and periodontal diseases [93]. Modulating bacterial adhesion to the surface of dental biomaterials through the formation of SP can disrupt the microbiota’s balance, or dysbiosis, on the material surface [94].

Within seconds, salivary proteins and other biomolecules rapidly adhere to oral surfaces, forming a layer that grows to approximately 10–20 nm thick after one minute and reaches around 1300 nm after 24 h [95]. The ultrastructural appearance of the pellicle layer exhibits a significant level of resemblance across various types of surfaces. Nevertheless, noticeable variations in the ultrastructural arrangement and thickness of the pellicle layer developed on the buccal and lingual sides were observed.

An important physicochemical consequence of salivary pellicle formation is the transformation of surface properties. Upon protein adsorption, the character of a surface—whether it is the oral mucosa or a biomaterial [96,97]—tends to shift from hydrophobic to more hydrophilic. This change is driven by the molecular composition of the adsorbed layer, particularly the presence of glycoproteins and mucins with strong water-binding capabilities. The alteration in surface wettability can significantly affect subsequent protein adhesion, bacterial colonization, and lubrication, ultimately influencing the performance and biocompatibility of dental materials in the oral environment.

The findings suggest that the formation of a salivary pellicle is primarily influenced by the presence of salivary biopolymers and the local shearing forces rather than being determined by the properties of the materials involved [98]. The dynamic nature of the adsorption process leads to conformational changes in proteins that were initially adsorbed, as subsequent proteins are adsorbed [99].

The adsorption process of proteins is primarily influenced by non-covalent interactions between the surfaces and molecules already sorbed on the surfaces. These interactions include hydrophobic, electrostatic forces, hydrogen bonding, and van der Waals forces [97]. Fischer and Aparicio [100] propose that this intricacy leads to the emergence of the “Vroman effect,” which characterizes the temporal alterations of proteins adsorbed from blood onto surfaces [101]. However, this phenomenon can also be extended to other systems, wherein smaller proteins with lower molecular weights are substituted by larger proteins with slower diffusion rates and higher molecular weights. The molecular shape also influences the adsorption dynamics. For instance, globular proteins possess a higher capacity to modify their shape to optimize surface interactions than rod-like proteins. Consequently, globular proteins exhibit a strong attraction towards surfaces due to their ability to maximize surface interactions [102]. An interesting consequence of this effect is that protein concentration in a solution does not always correlate with its concentration on the surface, as demonstrated for blood with albumin and fibrinogen [103], but can also be adapted to saliva [100].

There are two main ways in which SP adsorption occurs. Initially, the adsorption is based on the material–protein interactions. Later on, additional proteins adsorb onto the early proteins. These two phases result in the formation of two distinct layers of SP [104] (Figure 1). The layer that forms directly on the material is thinner and denser, while the layer on top is thicker and less dense. The lower layer is in direct contact with the material and typically has a network structure. On the other hand, the outer layer has a more varied structure with protein aggregates. The lower layer contains a higher concentration of proteins rich in proline, statins, histatins, and heavily glycosylated mucins than the outer layer. Interestingly, the lower layer is more resistant to mechanical wear than the outer layer. This is because the non-covalent interactions between the material and early proteins in the lower layer are more durable than those between early and late proteins in the outer layer [105].

The role of water is crucial in the process of SP adsorption. Water is the first molecule to adsorb onto the surface because it is much smaller than biomolecules. Therefore, proteins must displace the highly structured adsorbed layer of water to adsorb onto the surface [106]. It is suggested that other factors, such as temperature, pH, ionic strength of the solution, enzymatic cross-linking, and proteolysis of adsorbed molecules, may also affect the adsorption and structure of SP.

## 5. Mucoadhesion

In patients with xerostomia, the formation of the salivary pellicle—a natural protective layer that supports lubrication and microbial balance—is impaired. An ideal oral gel should therefore partially replicate its function, not only by moisturizing and lubricating but also by adhering to the mucosal surface.

In this context, rheological and tribological properties of gels converge in the essential function of mucoadhesion. This feature enables the gel to remain at the site of application, resist mechanical displacement, and provide sustained therapeutic effects.

### 5.1. Theories and Mechanisms of Mucoadhesion

Mucoadhesion refers to the ability of a substance, typically a polymer, to bind to mucosal surfaces via hydrogen bonding, van der Waals forces, or polymer chain interactions [107]. It plays a key role in ensuring that oral gels stay in place long enough to be effective, especially in the dynamic environment of the mouth.

Several theories have been proposed to explain the mechanisms behind mucoadhesion, offering insight into how oral gels interact with the complex environment of the oral cavity [108,109,110,111]. One of the most widely cited is the wetting theory, which posits that the effectiveness of adhesion is directly linked to how well the material spreads across and wets the mucosal surface. In the case of oral gels, the lower the contact angle and the higher the surface energy, the more intimate the contact with the mucosa—an essential first step for achieving strong adhesion. Another approach, the fracture theory, focuses on the mechanical force required to separate an adhesive material from the mucosal surface. Although this theory is particularly useful for understanding detachment forces and evaluating the strength of mucoadhesive bonds, it does not account for the molecular interpenetration between polymers and mucins, which is especially relevant in gel-based systems. The diffusion–interlocking theory addresses this limitation by suggesting that mucoadhesion results from the interpenetration and entanglement of polymer chains within the gel and mucin glycoproteins of the mucus layer. This mechanism depends heavily on the flexibility and molecular weight of the polymer, the duration of contact, and the hydration state of the formulation—all of which are critical design considerations for oral gels targeting xerostomic conditions.

On a more molecular level [112], the electronic theory proposes that mucoadhesion is partially driven by differences in electronic charge between the polymer and mucosal surface, resulting in electrostatic attractions. Though less commonly applied in gel systems, it provides insight into how charge distribution can influence initial contact and stability. Closely related is the adsorption theory, which explains mucoadhesion through the formation of secondary chemical bonds—such as hydrogen bonds and van der Waals forces—between the polymer and the mucosal tissue. These interactions are key to maintaining long-term adhesion, particularly in the constantly moving and hydrated oral environment. Finally, the dehydration theory suggests that when a highly hydrophilic polymer comes into contact with the mucus layer, water migrates from the mucus to the polymer, resulting in a localized dehydration effect. This dehydration enhances adhesion by increasing the physical proximity and interfacial contact between the two surfaces.

### 5.2. Evaluation Methods for Mucoadhesive Performance

To design effective oral gels for xerostomia, it is crucial not only to understand the mechanisms of mucoadhesion but also to select appropriate methods for its evaluation. These methods are generally classified into direct and indirect approaches, each offering different insights into the adhesive performance of mucoadhesive systems in the dynamic oral environment.

Direct methods involve the actual measurement of the interaction between the formulation and a biological or biomimetic mucosal surface. Common techniques include tensile force testing [113,114], residence time measurements [115,116], and force-based detachment assays [117,118], where the force or work required to separate a gel from the mucosa is used as an indicator of adhesive strength. These tests are often considered “functional” methods because they mimic the real mechanical challenges faced by a formulation in the oral cavity—such as saliva flow and tissue movement.

On the other hand, indirect methods evaluate the mucoadhesive potential based on physicochemical parameters that influence adhesion. These include assessments of hydration behavior, swelling index [119,120], contact angle [121], and viscosity [122]. While these approaches do not measure adhesion per se, they are valuable for predicting how a material might perform once in contact with the mucosa.

One of the most powerful and versatile indirect methods—which is also often used to complement direct testing—is rheological analysis. Originally introduced in the early 1990s and still widely used today, the rheological approach provides a mechanistic understanding of mucoadhesive interactions by analyzing changes in a formulation’s viscosity and viscoelastic properties upon mixing with mucin.

The concept is grounded in the assumption that mucoadhesion involves chain entanglement and physicochemical bonding between the polymer and mucin molecules. As Rossi et al. [123] explain, when such interactions occur, they result in measurable changes in the rheological profile of the mixture. One of the key indicators is the rheological synergism, where the observed viscosity of the polymer–mucin mixture exceeds the sum of the viscosities of the individual components. This deviation is considered evidence of mucoadhesive bonding strength. To quantify this, the viscosity of the mixed system (ηₜ) is compared to the theoretical additive value: η_t_ = η_m_ + η_p_ + η_b_, where η_m_ is the viscosity of the mucin solution, η_p_ that of the polymer, and η_b_ is attributed to bioadhesive interactions. A positive value of η_b_—i.e., when the mixture is more viscous than expected—indicates the formation of a mucoadhesive joint.

In addition to viscosity measurements, viscoelastic analysis offers deeper insight into the structural behavior of polymer–mucin systems. In this method, the formulation is exposed to oscillatory shear stress, and its response is characterized by two parameters: storage modulus (G′) and loss modulus (G″). These describe the material’s elastic and viscous components, respectively—G′ indicating the ability to store energy and behave like a solid, and G″ reflecting the tendency to dissipate energy as a viscous fluid.

Mucoadhesion is inferred when the viscoelastic response of the mixture exceeds that of the individual components, quantified by calculating the differential moduli (ΔG′ and ΔG″). A positive deviation—i.e., ΔG′ > 0 and ΔG″ > 0—indicates that a structured and cohesive network has formed between the polymer and mucin, consistent with the establishment of mucoadhesive interactions.

These changes are not merely numerical; they reflect real physical entanglements and secondary bonding at the interface. For oral gels, where retention and stability are critical, such rheological signals provide robust evidence that the formulation can maintain intimate, prolonged contact with the oral mucosa—even in the challenging conditions associated with xerostomia.

What makes rheological testing particularly attractive is its simplicity, reproducibility, and sensitivity. It is a non-destructive method that can be performed with minimal sample volumes and under controlled conditions that simulate the oral environment. Moreover, recent advancements—such as creep tests and model-based compliance analysis—have enabled deeper exploration of how gel structure and interfacial bonding evolve over time.

However, it is important to acknowledge the limitations. Rheological results may vary depending on the type and preparation of mucin used, and cross-comparison of polymers with different chemical structures should be approached with caution. For this reason, Rossi and colleagues [123] recommend using rheological analysis as a mechanistic tool, ideally paired with performance-based methods like retention testing on inclined planes, particularly when assessing mucoadhesive gels for xerostomic patients.

## 6. Impact of Lubrication on Oral Biomechanics and Usage of Prosthetic Devices

### 6.1. Role of Lubrication in Maintaining Oral Biomechanical Balance

The stomatognathic system, encompassing the intricate interplay of oral tissues and organs, is fundamental to vital functions such as mastication, speech, respiration, and emotional expression. This complex functional unit has evolved over millions of years, adapting to physiological stresses and adhering to biomechanical principles [124]. Its anatomical and functional integrity is key to maintaining oral homeostasis. However, disruptions—such as tooth loss—can disturb this balance, necessitating restorative interventions. Similarly, inadequate lubrication may impair the mechanical harmony of the system and requires appropriate management.

Interestingly, it is only in the relatively recent history of prosthetic dentistry that we have come to understand dentures as foreign bodies within the oral cavity—entities that the stomatognathic system may tolerate to varying degrees [125]. Each patient responds individually to prosthetic rehabilitation, depending on both local tissue conditions and systemic adaptability.

The loss of teeth and xerostomia (dry mouth) are closely linked. Hence, it is crucial to ensure proper lubrication of prosthetic surfaces. The stomatognathic system can adjust to changes, which means that even a significant loss of occlusal tooth substance due to wear may not necessarily affect oral health. However, rapid loss of tooth substance or vertical height can adversely affect the function of the teeth and the temporomandibular joint. There is a lack of scientific data regarding the risk to the stomatognathic system resulting from dental restorations with higher wear than enamel. However, when material loss becomes clinically visible, wear affects the aesthetic appearance of the restoration, particularly in the anterior region. Since there is no scientific evidence that more significant wear of restorative materials may be associated with possible side effects, it can be concluded that wear is primarily an aesthetic problem and may potentially limit masticatory function.

The impact of diminished lubrication on the biomechanical behavior of oral soft tissues presents a compelling area of inquiry. The oral mucosa fulfills a critical physiological function by distributing masticatory forces and safeguarding the underlying residual ridge from excessive loading [126]. As a highly vascularized tissue, the mucosa possesses a substantial volume of interstitial fluid, contributing to its protective capacity through a mechanism of mechanical cushioning. However, the pressure exerted by dentures induces a pumping effect that displaces interstitial fluid towards adjacent unloaded tissues. This fluid movement compels collagen fibers to align along the axes of mechanical stress, affording passive protection to the connective tissue and underlying bone. Increased masticatory loads result in a gradual elevation of interstitial fluid pressure. Upon exceeding vascular pressure, interstitial fluid pressure can compromise blood flow, potentially inducing localized ischemia. This time-dependent process is exacerbated by prolonged loading periods, ultimately reaching a plateau. Both the magnitude and duration of the applied load directly influence the extent of ischemia. Prolonged interference with blood flow culminates in local oxygen deprivation (anoxia) and the accumulation of metabolites, ultimately contributing to the deterioration of supporting bony structures, a condition clinically termed residual ridge resorption [127,128,129]. To mitigate these detrimental sequelae, oral gels should provide a supportive function while ensuring adequate mucosal hydration, thereby attenuating the adverse consequences of interstitial fluid displacement [130]. This necessitates the presence of appropriate viscoelastic properties, specifically elasticity and the capacity to accumulate deformation energy, without concomitant alteration of consistency during compression.

When a mechanical load is removed, the mucosa can recover [131]. Releasing surface pressure helps the interstitial fluid flow back, facilitating recovery. The time it takes for recovery is directly proportional to the magnitude and duration of the load [132]. Wearing dentures for more than 6 months can lead to long-lasting effects on the blood supply, decreasing blood flow recovery in the mucosa after compression release. Continuous clenching causes ischemia, which further delays the recovery of blood flow. Prolonged and constant pressure can cause changes in oral anatomy, affecting physiological responses [130].

### 6.2. Consequences of Reduced Lubrication for Denture Wearers

The oral mucosa is a protective barrier for the underlying residual ridge, enduring compressive forces and surface shear resulting from friction caused by dentures. It is widely recognized that wearing dentures can lead to the development of mucosal lesions [133]. These lesions can be attributed to microbial plaque on the dentures and the constituents of the denture materials and can manifest as acute or chronic reactions to mechanical injury. Many symptoms associated with denture-induced mucosal lesions, such as traumatic ulcers, angular cheilitis, irritation hyperplasia, and keratosis, are primarily caused by the frictional forces exerted on the oral mucosa. Unfortunately, these symptoms are often difficult to treat effectively [134,135,136].

It is crucial to comprehensively understand the dynamic interaction between the denture and the supporting mucosa to prevent any potential soft-tissue injuries [135]. Accurately determining the nonlinear elastic contact is essential for properly transmitting occlusal load. This interactive response is closely associated with the friction coefficient, which varies significantly among individuals due to their unique oral physiological conditions and the specific denture materials utilized [137].

The presence and quality of saliva influence the friction coefficient, which affects the contact conditions. The reduction in saliva production has been found to significantly impact the usage of dentures, often leading to membrane stomatitis. Experimental studies have demonstrated that “dried” surfaces, which simulate xerostomia with a hydration index close to 0, exhibit high friction coefficients ranging from 0.3 to 0.4 [138]. Conversely, well-lubricated conditions have been reported to have a low friction coefficient value of approximately 0.02 [138].

The friction coefficient can vary among denture materials, even when the oral condition remains unchanged. Materials with higher wettability tend to create a superior lubricating layer between the supporting mucosa surface and the denture base. This layer helps protect the surface tissue by reducing friction. In a study mentioned in the literature, seven common denture liner materials were tested in silico. When dry, the friction coefficient ranged from 0.35 to 0.97. However, after wetting in a warm water bath, the friction coefficient decreased from 0.24 to 0.90. It was observed that acrylic resin material exhibited significantly better wettability than silicones, drastically reducing the friction coefficient when wet [139,140].

This shows the importance of oral lubrication, especially in the context of people using prosthetic devices, and the need to develop new strategies for treating xerostomia.

## 7. Oral Lubrication, Tribology, and Rheology

### 7.1. Rheological and Tribological Principles in Oral Lubrication

Oral lubrication is gaining increasing recognition in biomedicine and personal care, specifically in treating dry mouth and developing oral care products [141]. It encompasses the various processes and mechanisms that minimize the frictional forces occurring in the oral interfaces between the teeth, tongue, palate, and mucosa. The lubrication of these surfaces can be achieved through the natural production of saliva, which acts as a bio-lubricant, or through the application of externally derived lubricants present in oral care products. The proper functioning of oral tissues, including activities such as eating, swallowing, speech, tactile perception, and the prevention of microbial adhesion, relies on the effectiveness of oral lubrication. With a deep understanding of the importance and relevance of oral lubrication research, we focus on delving into the critical aspects of the relationship between tribology (the science concerned with the principles of friction, lubrication, and wear of interacting surfaces in relative motion) and rheology (the study of the deformation and flow behavior of matter, particularly fluids and soft solids such as biological lubricants).

Most of the early studies in this field primarily focused on the wear (the progressive loss of material from a surface due to mechanical interactions such as friction or repeated contact) of the hardest surface that interacts, i.e., enamel [142], with the aim of innovating dental restorations and implants [143,144,145]. Mair [146] provided a basic overview of these issues, while Lambrechts et al. [147] presented an essential summary of how to simulate and predict wear in the oral cavity. Many modern literature reviews focus on explaining the wear mechanisms of hard materials such as metal alloys [148,149,150] and even extend these aspects to tribo-corrosion and bio-tribocorrosion [151,152]. However, in recent times, there has been a shift in the research focus towards a better understanding of oral lubrication [153,154,155], not only the wear of dental materials.

**Figure 2:**
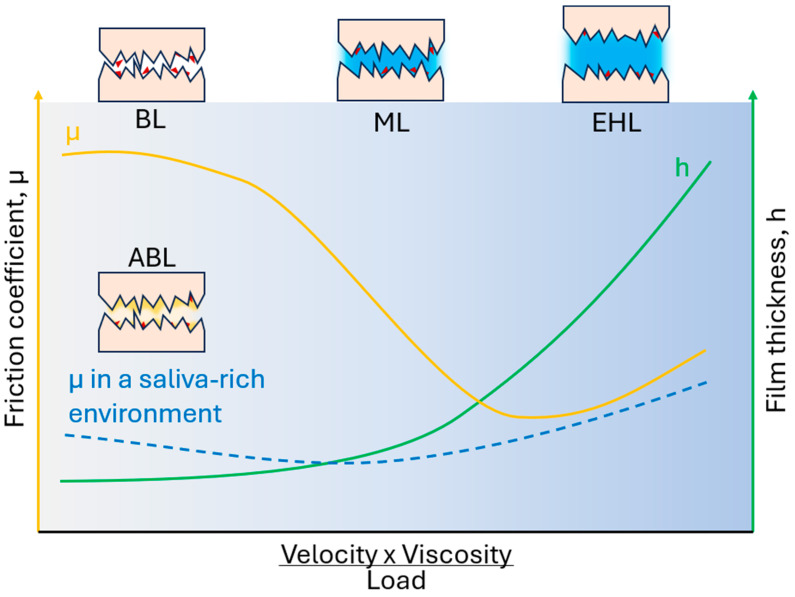
Stribeck curves display the friction coefficient as a function of velocity, the viscosity of lubricant, and load (adapted from Sarkar et al. [155]). According to classical theories of rheology, the coefficient of friction varies with the increase in the thickness of the lubricating film that separates the rubbing surfaces. It is at its highest value in the boundary lubrication (BL) regime, then decreases during mixed lubrication (ML), where it reaches a minimum, and finally slightly increases during elastohydrodynamic lubrication (EHL). When lubrication occurs in the oral cavity environment, a layer of amphiphilic, polar, brush-like macromolecules adsorbs onto the rubbing surfaces, significantly reducing the coefficient of friction. This phenomenon is known as aqueous boundary lubrication (ABL).

Understanding the forces that occur when oral surfaces move against each other in the presence of saliva or other oral fluids can be aided by considering classical rheology and tribology, as discussed by Sarkar et al. [155]. These concepts being deliberated pertain to the limitations arising from the proximity of surfaces. In the rheological limit, a fluid film completely separates the surfaces, with its thickness typically being one order of magnitude greater than the characteristic dimensions of individual components, such as the polymer radius of gyration in a solution or the size of microparticles in colloidal suspensions. At this stage, interphase boundaries and surface properties become less significant. To investigate the relationship between the measured forces and macroscopic deformation, lubricants can be subjected to various shear conditions using rheological techniques. Furthermore, by analyzing rheological data using appropriate constitutive equations or microscopic models, one can obtain information about the composition and structure of the lubricant. On the other side, tribology focuses on the interaction between surfaces that are close to each other. It encompasses three distinct regimes, which range from direct or partial contact between surfaces to the separation of surfaces by a continuous layer of fluid. These regimes are traditionally described by Stribeck curves, which illustrate the friction coefficient as a function of various operating conditions, including load, surface speed, and lubricant viscosity (Figure 2). In regimes where a continuous fluid layer separates contact surfaces, the rheological properties of the lubricant play a crucial role. However, the tribological limit includes thin fluid films with a thickness comparable to the surface roughness and, in consequence, investigates shear rates at least 10 times higher than the maximum shear rate of saliva typically reported in the literature and measured by rheological techniques. The thickness of the film is determined by the balance between the contact load and the increased hydrodynamic pressure exerted by the fluid. This pressure is influenced by fluid viscosity, surface velocity, and contact geometry. Unlike rheology, which focuses on the flow behavior of fluids, tribology examines how the presence or absence of fluid and its interaction with contact surfaces influence friction forces and their behavior under different operating conditions. Oral lubrication encompasses both rheological and tribological aspects. Within the oral cavity, saliva, a viscoelastic and highly adhesive natural lubricant, plays a crucial role in lubrication across three different regimes within the Stribeck curve.

The friction coefficient demonstrates limited or no dependence on surface dynamics in the boundary lubrication regime, commonly observed at lower working speeds. This regime prevails during high-load and low-velocity periods with negligible hydrodynamic forces. Consequently, the contact zone lacks a sufficient amount of lubricant. Friction forces in this regime are primarily influenced by surface properties, such as viscoelasticity (soft surfaces with high stiffness are characterized by a lower coefficient of boundary friction than those with low stiffness) [64,156], roughness (in situ friction coefficient was significantly positively correlated with tongue-surface roughness) [157], and interactions of a surface-bound film [64], rather than the rheology of the confined lubricating fluid. It is essential to consider the complex features of the different biological surfaces involved to understand the lubrication processes in the boundary regime. These surfaces include various soft tissues arranged in different configurations, such as hard-soft (between the hard palate and the tongue) or soft-soft (between the tongue and the soft palate), which are lubricated by saliva and other mucosal lubricants.

### 7.2. Biological Interfaces, Salivary Films, and Friction Reduction Mechanisms

Different materials are employed to investigate friction processes within the oral cavity, aiming to replicate natural structures to a certain extent. Hard tissues predominantly originate from natural sources, either human or animal [158], or are replicated using ceramic materials like aluminum oxide [159]. Soft tissues are substituted with animal counterparts, such as pig tissues [160], or are imitated using PDMS [161]. Nevertheless, it is crucial to consider several aspects concerning the properties of oral cavity tissues, using the tongue as an illustrative example.

The tongue’s surface is not smooth and has various embedded papillae, which cause roughness in different areas. Most of the tongue’s surface is covered by filiform papillae, which are 420–500 μm in diameter at the root and have a height of 250 μm [162]. The tongue’s roughness, measured by the R_a_ value, is smaller than expected when measured using a lingual impression with silicon dental material, ranging from 42.5 to 101.4 μm, due to plastic deformation during the impression process. Despite its roughness, the tongue is not coarse due to its reduced “stiffness.” When measured in vivo by magnetic resonance elastography, the shear storage modulus of the tongue and soft palate is 2.67 and 2.53 kPa, respectively [163]. However, during intense tongue movements such as speaking or eating, the stiffness of the tongue may increase to 30–70 kPa due to vascularization and the increase in stiffness under the influence of rapid deformations [164]. The tongue is keratinized and can be intrinsically hydrophobic and weakly polar. However, when wetted by saliva, the tongue surface becomes hydrophilic [165]. Saliva contains 99% water and <1% organic/inorganic compounds, which have material properties far from water. It can be classified into a fluid-like bulk saliva and an adsorbed salivary pellicle. Salivary molecules adhere to the oral surfaces and help maintain an adsorbed salivary film [97]. Loss of saliva pellicle may result in poor surface hydration, reduced wettability, and, therefore, higher hydrophobicity of the tongue surface.

A significant decrease in the friction coefficient can be observed when the contacting oral surfaces are coated with an amphiphilic macromolecule with polar head groups. This phenomenon, known as aqueous boundary lubrication, has been extensively studied due to its relevance in physiological processes [153]. Several research studies have explored the structure and composition of adsorbed salivary films and their correlation with lubrication properties. The coefficient of interface friction (μ) (the ratio of the tangential force resisting motion to the normal force) between smooth surfaces lubricated with human whole-mouth saliva (WHS) has been reported to range between 0.01 and 0.1 [166,167,168,169]. The experimental conditions can affect the values of μ, but they are generally higher for diluted saliva samples. The minimum values of μ obtained are at least two orders of magnitude lower than those observed under dry friction or with water between hydrophobic surfaces. The proteins that form the film can directly bind to the tooth enamel or mucosal epithelial layer. The composition and structure of the salivary pellicle (SP) are complex and depend on the type of substrate (teeth, keratinized, or non-keratinized soft tissue) to which it adheres and on its location in the oral cavity [97]. It is not precisely known which salivary proteins are responsible for effective lubrication. Mucins, proline-rich proteins (PRP), and statherin are suggested to be the most significant among them. The evaluation of mucins under neutral pH conditions (as in saliva) showed that their lubrication ability between hydrophobic surfaces does not fall below a friction coefficient of 0.1 [170]. However, it should be noted that lubricating properties may be sensitive to the structure of the mucin layer, which may not always be reflected using purified mucins, so these results must be treated cautiously. In some specific conditions, such as low pH of 2 for porcine gastric mucins, boundary friction coefficients around 0.02 were observed [171]. Early works by Douglas et al. [172] focused on lubrication between polished enamel surfaces using saliva and saliva fractions. It was found that purified statherin from saliva gave a lower friction coefficient (less than 0.4) than the mucin-rich fraction (less than 0.7). The hypothesis of the crucial role of statherin was analyzed, and it turned out that purified statherin has a relatively marginal ability to lubricate on non-enamel substrates [173]. The same studies showed that friction coefficients could be even lower for purified PRP than for WHS. However, in studies for physiological concentrations of PRP without mucins, these friction coefficients were relatively high (above 0.24) [166].

Considering these aspects, Yakubov et al. [104] conducted a thorough analysis of the lubrication of oral salivary proteins and arrived at several fundamental conclusions. The SP consists of two functional layers (not necessarily spatial) (Figure 1): a detergent-resistant, non-lubricating layer composed of small proteins with low molecular weight and a detergent-sensitive, hydrated layer rich in mucins, which provides lubrication. This layer has a brush-like structure, and it has been demonstrated that the concentration of high molecular weight mucins is crucial for achieving adequate lubrication. Low molecular weight proteins are necessary to acquire sufficient wear resistance. The addition of PRP proteins has a positive effect on reducing the friction coefficient. Cystatin and statherin facilitate the adhesion of the forming SP to hydrophobic surfaces in the early stages.

Returning to the Stribeck curve (Figure 2), in a mixed lubrication regime, the friction coefficient decreases as the ratio of hydrodynamic forces to contact load increases. As this ratio increases, more lubricant is drawn into the contact area, reducing the effective contact between surface asperities and, thus, the friction force. The lowest values of friction coefficients are commonly observed in the mixed regime at the border with the hydrodynamic regime, which signifies the transition between partially (interrupted by a liquid layer) and completely separated surfaces by a liquid layer. In the latter case, a thin layer of lubricant entirely sustains the contact load, maintaining the separated surfaces that do not directly contact each other. It should be noted that saliva and most saliva substitutes exhibit non-Newtonian behavior, with their viscosity decreasing as the shear rate increases. A low friction coefficient can be maintained through the appropriate viscosity of the lubricant as well as the flexibility and stability of the lubricating film. Lubrication in these regimes most often occurs during food processing in the oral cavity. The rheological properties of food largely shape its perceived texture [174].

Comprehensive approaches that consider tribological and rheological aspects are rarely applied when researching oral hygiene products, including oral gels. Known solutions based on glycerin, oils, or polysaccharides, which mimic natural structures to a limited extent, are commonly used in formulation development. Furthermore, most products designed to treat xerostomia symptomatically fail to replicate the function of the acquired pellicle, particularly the mechanisms of boundary lubrication, which rely on amphiphilic molecules.

### 7.3. Rheological Evaluation of Commercial Oral Gels

The management of xerostomia, particularly in denture wearers, requires more than symptomatic relief. As this review has demonstrated, saliva plays a fundamental role not only in maintaining mucosal hydration and barrier function but also in enabling effective lubrication and mechanical buffering under prosthetic devices. Most currently available oral gels, while offering temporary comfort, fail to reproduce the full spectrum of salivary functions—particularly those associated with mucin-mediated hydration, mucoadhesion, and tribological performance.

Although various strategies to restore or mimic salivary function—such as mucin analogs, polymeric mimetics, or mucoadhesive hydrogels—have been explored, their clinical implementation remains limited. A key issue is the lack of standardized performance metrics that would allow clinicians to select formulations based on their physicochemical or mechanical behavior. In contrast to fields such as lubrication engineering, where viscosity grading systems (e.g., SAE classifications for engine oils) support evidence-based product matching, oral gels lack analogous functional labeling. This absence is notable given that viscosity, elasticity, and mucoadhesive properties are directly linked to therapeutic outcomes in xerostomia management.

**Figure 3:**
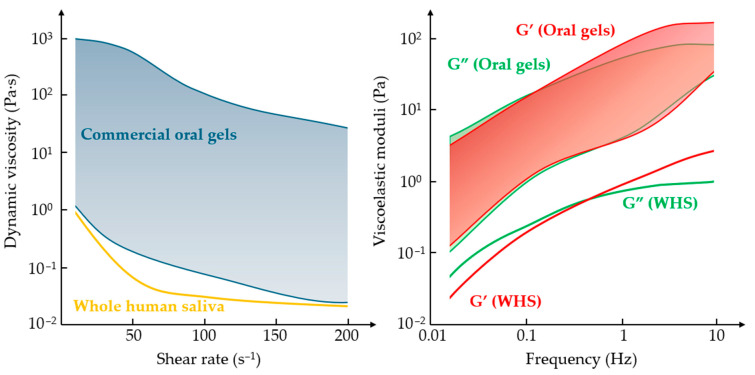
Rheological comparison of whole human saliva (WHS) and selected commercial oral gels. Left: dynamic viscosity as a function of shear rate, showing the pronounced shear-thinning of WHS compared to a more gradual decrease in gels. Right: viscoelastic moduli (G′ and G″) as functions of oscillatory frequency, illustrating the greater structural rigidity and elastic dominance of gels relative to WHS. These differences reflect the gels’ need for retention and mechanical durability but highlight their limitations in replicating the dynamic responsiveness of saliva. Measurements were conducted at 37 °C using a cone-plate geometry for viscosity testing and a parallel-plate geometry for viscoelastic characterization, across physiologically relevant shear rates and oscillation frequencies.

To address this gap, we performed a rheological analysis of several commercially available oral gels widely accessible over the counter in Poland (Figure 3). Although the formulations varied only modestly in composition—typically based on cellulose derivatives (carboxymethylcellulose, carboxyethylcellulose, hydroxyethylcellulose), carbomer, carrageenan, glycerol, and essential oils—the exact concentrations were not disclosed by the manufacturers. Based on consistency and usage instructions, these gels are designed for localized application by spreading over oral tissues.

All tested formulations exhibited shear-thinning behavior, characterized by a decrease in dynamic viscosity with increasing shear rate. This property is fundamental for oral functionality: it allows gels to maintain positional stability at rest while enabling ease of movement during speaking or chewing, when shear forces increase. WHS, serving as the physiological reference, also displays marked shear-thinning behavior—its viscosity drops rapidly even under modest shear. In contrast, oral gels are substantially more viscous and demonstrate a slower, more gradual decline in viscosity across three logarithmic orders. This heightened viscosity improves gel retention but also reflects the fact that, unlike saliva, gels are not continuously replenished and must resist mechanical clearance by food and speech.

Saliva’s rheological profile is not solely defined by viscosity. Its viscoelastic behavior—combining fluid and elastic responses—is essential for forming a stable, load-bearing film under dentures. In our analysis, WHS showed relatively balanced viscoelastic properties, with the elastic (G′) and viscous (G″) moduli remaining close across low deformation frequencies. At higher frequencies—corresponding to more rapid mechanical strain—G′ surpassed G″, suggesting an increased ability to transiently resist deformation, much like the brief resistance experienced when striking the surface of water.

The gels tested also exhibited viscoelasticity, but with significantly different profiles. At low frequencies, they were primarily viscous, similar to saliva. However, at higher frequencies, they became increasingly elastic, with G′ often exceeding G″ by an order of magnitude—indicating a much stiffer, more solid-like behavior. While this may enhance durability and adherence in the moist and dynamic oral environment, it also underscores their limited ability to replicate the subtle, adaptable mechanics of natural saliva.

These findings are summarized in Figure 3, which compares the shear-dependent viscosity and viscoelastic moduli of WHS and commercial oral gels. The results clearly highlight functional discrepancies between natural saliva and synthetic formulations, emphasizing the need for oral gels to be evaluated and labeled based on properties such as viscosity range, shear-thinning behavior, and viscoelastic balance.

To move beyond the limitations of current formulations, there is a clear need for next-generation oral gels that more faithfully replicate the biophysical and functional features of native saliva. Ideally, these gels should combine controlled viscoelasticity with strong mucoadhesive properties and a molecular architecture that mimics the amphiphilic character of salivary mucins. This includes incorporating both hydrophobic and highly glycosylated hydrophilic domains, capable of forming persistent, hydrated boundary layers on mucosal and prosthetic surfaces alike.

Moreover, gels should be optimized to work synergistically with residual saliva and the salivary pellicle, not merely act as passive coatings. The integration of functional biomimetic components—such as synthetic glycopolymers, modular mucin mimetics, or rationally designed amphiphilic macromolecules—could provide a major leap forward. Additionally, formulation design must consider the tribological realities of the oral cavity: high-frequency deformation, interfacial shear, and constant hydration challenge.

Equally important is the establishment of quantifiable performance standards. Introducing classification schemes that reflect critical physical parameters—viscosity ranges under oral shear conditions, elastic response profiles, and hydration stability—could transform both product development and clinical decision-making. Much like viscosity grades guide lubricant selection in engineering, standardized rheological and tribological benchmarks would allow clinicians to match gel properties to patient-specific needs and usage contexts.

In short, the future of xerostomia treatment lies not in more products but in smarter, data-driven formulations that integrate biomimicry, mechanical performance, and clinical relevance.

## Data Availability

The raw data (rheological measurements) supporting the conclusions of this article will be made available by the authors on request.

## References

[1] Humphrey S.P., Williamson R.T. (2001). A Review of Saliva: Normal Composition, Flow, and Function. J. Prosthet. Dent..

[2] Levine M.J., Aguirre A., Tabak L.A., Hatton M.N. (1987). Artificial Salivas: Present and Future. J. Dent. Res..

[3] Han Y., Jia L., Zheng Y., Li W. (2018). Salivary Exosomes: Emerging Roles in Systemic Disease. Int. J. Biol. Sci..

[4] Dawes C., Pedersen A.M.L., Villa A., Ekström J., Proctor G.B., Vissink A., Aframian D., McGowan R., Aliko A., Narayana N. (2015). The Functions of Human Saliva: A Review Sponsored by the World Workshop on Oral Medicine VI. Arch. Oral Biol..

[5] Oudhoff M.J., Bolscher J.G.M., Nazmi K., Kalay H., van’t Hof W., Amerongen A.V.N., Veerman E.C.I. (2008). Histatins Are the Major Wound-Closure Stimulating Factors in Human Saliva as Identified in a Cell Culture Assay. FASEB J..

[6] Torres P., DÍaz J., Arce M., Silva P., Mendoza P., Lois P., Molina-Berríos A., Owen G.I., Palma V., Torres V.A. (2017). The Salivary Peptide Histatin-1 Promotes Endothelial Cell Adhesion, Migration, and Angiogenesis. FASEB J..

[7] Guo S., DiPietro L.A. (2010). Critical Review in Oral Biology & Medicine: Factors Affecting Wound Healing. J. Dent. Res..

[8] Vila T., Rizk A.M., Sultan A.S., Jabra-Rizk M.A. (2019). The Power of Saliva: Antimicrobial and Beyond. PLoS Pathog..

[9] Shakeeb N., Varkey P., Ajit A. (2021). Human Saliva as a Diagnostic Specimen for Early Detection of Inflammatory Biomarkers by Real-Time RT-PCR. Inflammation.

[10] Scully C. (2014). Challenges in Predicting Which Oral Mucosal Potentially Malignant Disease Will Progress to Neoplasia. Oral Dis..

[11] Kim Y.J. (2023). Xerostomia and Its Cellular Targets. Int. J. Mol. Sci..

[12] Adolfsson A., Lenér F., Marklund B., Mossberg K., Çevik-Aras H. (2022). Prevalence of Dry Mouth in Adult Patients in Primary Health Care. Acta Odontol. Scand..

[13] Dibello V., Zupo R., Sardone R., Lozupone M., Castellana F., Dibello A., Daniele A., De Pergola G., Bortone I., Lampignano L. (2021). Oral Frailty and Its Determinants in Older Age: A Systematic Review. Lancet Healthy Longev..

[14] Niedermeier W., Huber M., Fischer D., Beier K., Müller N., Schuler R., Brinninger A., Fartasch M., Diepgen T., Matthaeus C. (2000). Significance of Saliva for the Denture-Wearing Population. Gerodontology.

[15] Al-Dwairi Z., Lynch E. (2014). Xerostomia in Complete Denture Wearers: Prevalence, Clinical Findings and Impact on Oral Functions. Gerodontology.

[16] Bergdahl M. (2000). Salivary Flow and Oral Complaints in Adult Dental Patients. Community Dent. Oral Epidemiol..

[17] Lakhyani R., Wagdargi S.S. (2012). Saliva and Its Importance in Complete Denture Prosthodontics. Natl. J. Integr. Res. Med..

[18] Lal Q., Godil A., Shaikh M., Musani S., Dugal R., Kirad A. (2023). Wettability of Two Different Artificial Saliva Substitutes on Injection Moulded Heat Polymerized Acrylic Resin and CAD-CAM Acrylic Resin: An In Vitro Study. Dent. 3000.

[19] Taqa A.A., Nazhat M.N., Basshi T.Y., Al_jader G.H. (2018). Evaluation of Physical and Chemical Properties of Saliva on Retention of Complete Denture (In Vitro Study). Int. J. Dent. Res..

[20] Narita T., Qi B., Murakami M., Sugiya H. (2019). Pilocarpine Induces the Residual Secretion of Salivary Fluid in Perfused Submandibular Glands of Rats. PLoS ONE.

[21] Yasuda H., Niki H. (2002). Review of the Pharmacological Properties and Clinical Usefulness of Muscarinic Agonists for Xerostomia in Patients with Sjögren’s Syndrome. Clin. Drug Investig..

[22] Rocchi C., Barazzuol L., Coppes R.P. (2021). The Evolving Definition of Salivary Gland Stem Cells. NPJ Regen. Med..

[23] Bianco P., Cao X., Frenette P.S., Mao J.J., Robey P.G., Simmons P.J., Wang C.Y. (2013). The Meaning, the Sense and the Significance: Translating the Science of Mesenchymal Stem Cells into Medicine. Nat. Med..

[24] Lombaert I.M.A., Brunsting J.F., Weirenga P.K., Faber H., Stokman M.A., Kok T., Visser W.H., Kampinga H.H., de Haan G., Coppes R.P. (2008). Rescue of Salivary Gland Function after Stem Cell Transplantation in Irradiated Glands. PLoS ONE.

[25] Song W., Liu H., Su Y., Zhao Q., Wang X., Cheng P., Wang H. (2024). Current Developments and Opportunities of Pluripotent Stem Cells-Based Therapies for Salivary Gland Hypofunction. Front. Cell Dev. Biol..

[26] Baum B.J., Alevizos I., Zheng C., Cotrim A.P., Liu S., McCullagh L., Goldsmith C.M., Burbelo P.D., Citrin D.E., Mitchell J.B. (2012). Early Responses to Adenoviral-Mediated Transfer of the Aquaporin-1 CDNA for Radiation-Induced Salivary Hypofunction. Proc. Natl. Acad. Sci. USA.

[27] Rocchi C., Emmerson E. (2020). Mouth-Watering Results: Clinical Need, Current Approaches, and Future Directions for Salivary Gland Regeneration. Trends Mol. Med..

[28] Darvell B.W., Clark R.K.F. (2000). The Physical Mechanisms of Complete Denture Retention. Br. Dent. J..

[29] Fábián T.K., Hermann P., Beck A., Fejérdy P., Fábián G. (2012). Salivary Defense Proteins: Their Network and Role in Innate and Acquired Oral Immunity. Int. J. Mol. Sci..

[30] Sotres J., Pettersson T., Lindh L., Arnebrant T. (2012). NanoWear of Salivary Films vs. Substratum Wettability. J. Dent. Res..

[31] Engel A.S., Kranz H.T., Schneider M., Tietze J.P., Piwowarcyk A., Kuzius T., Arnold W., Naumova E.A. (2020). Biofilm Formation on Different Dental Restorative Materials in the Oral Cavity. BMC Oral Health.

[32] Kolenbrander P.E., Palmer R.J., Periasamy S., Jakubovics N.S. (2010). Oral Multispecies Biofilm Development and the Key Role of Cell-Cell Distance. Nat. Rev. Microbiol..

[33] Chibly A., Aure M., Patel V., Hoffman M.P. (2022). Salivary Gland Function, Development, and Regeneration. Physiol. Rev..

[34] Pedersen A.M.L., Sørensen C.E., Proctor G.B., Carpenter G.H., Ekström J. (2018). Salivary Secretion in Health and Disease. J. Oral Rehabil..

[35] Proctor G.B. (2016). The Physiology of Salivary Secretion. Periodontol. 2000.

[36] Song E.A.C., Chung S.H., Kim J.H. (2024). Molecular Mechanisms of Saliva Secretion and Hyposecretion. Eur. J. Oral Sci..

[37] Saleh J., Figueiredo M.A.Z., Cherubini K., Salum F.G. (2015). Salivary Hypofunction: An Update on Aetiology, Diagnosis and Therapeutics. Arch. Oral Biol..

[38] Mercadante V., Jensen S.B., Smith D.K., Bohlke K., Bauman J., Brennan M.T., Coppes R.P., Jessen N., Malhotra N.K., Murphy B. (2021). Salivary Gland Hypofunction and/or Xerostomia Induced by Nonsurgical Cancer Therapies: ISOO/MASCC/ASCO Guideline. J. Clin. Oncol..

[39] Murray Thomson W., Poulton R., Mark Broadbent J., Al-Kubaisy S. (2006). Xerostomia and Medications among 32-Year-Olds. Acta Odontol. Scand..

[40] Tanasiewicz M., Hildebrandt T., Obersztyn I. (2016). Xerostomia of Various Etiologies: A Review of the Literature. Adv. Clin. Exp. Med..

[41] Xiao F. (2017). Neuromyotonia as an Unusual Neurological Complication of Primary Sjögren’s Syndrome: Case Report and Literature Review. Clin. Rheumatol..

[42] Wang X., Hu C., Eisbruch A. (2011). Organ-Sparing Radiation Therapy for Head and Neck Cancer. Nat. Rev. Clin. Oncol..

[43] Glennon S.G., Huedo-Medina T., Rawal S., Hoffman H.J., Litt M.D., Duffy V.B. (2019). Chronic Cigarette Smoking Associates Directly and Indirectly with Self-Reported Olfactory Alterations: Analysis of the 2011–2014 National Health and Nutrition Examination Survey. Nicotine Tob. Res..

[44] Diep M.T., Jensen J.L., Skudutyte-Rysstad R., Young A., Sødal A.T.T., Petrovski B.É., Hove L.H. (2021). Xerostomia and Hyposalivation among a 65-Yr-Old Population Living in Oslo, Norway. Eur. J. Oral Sci..

[45] Millsop J.W., Wang E.A., Fazel N. (2017). Etiology, Evaluation, and Management of Xerostomia. Clin. Dermatol..

[46] Fathi Y., Hoseini E.G., Atoof F., Mottaghi R. (2021). Xerostomia (Dry Mouth) in Patients with COVID-19: A Case Series. Future Virol..

[47] Huang N., Pérez P., Kato T., Mikami Y., Okuda K., Gilmore R.C., Conde C.D., Gasmi B., Stein S., Beach M. (2021). SARS-CoV-2 Infection of the Oral Cavity and Saliva. Nat. Med..

[48] Weng P.L., Aure M.H., Maruyama T., Ovitt C.E. (2018). Limited Regeneration of Adult Salivary Glands after Severe Injury Involves Cellular Plasticity. Cell Rep..

[49] Bossola M., Tazza L. (2012). Xerostomia in Patients on Chronic Hemodialysis. Nat. Rev. Nephrol..

[50] Mody M.D., Rocco J.W., Yom S.S., Haddad R.I., Saba N.F. (2021). Head and Neck Cancer. Lancet.

[51] Siegel R.L., Kratzer T.B., Giaquinto A.N., Sung H., Jemal A. (2025). Cancer Statistics, 2025. CA Cancer J. Clin..

[52] Hacioglu M.B., Erdogan B., Bardakcı M., Algın E., Gulbagcı B., Hacibekiroglu I., Hamdard J., Olmez O.F., Akkus H., Oksuzoglu B. (2023). Major and Minor Salivary Gland Cancers: A Multicenter Retrospective Study. Head Neck.

[53] Nutting C.M., Morden J.P., Harrington K.J., Urbano T.G., Bhide S.A., Clark C., Miles E.A., Miah A.B., Newbold K., Tanay M.A. (2011). Parotid-Sparing Intensity Modulated versus Conventional Radiotherapy in Head and Neck Cancer (PARSPORT): A Phase 3 Multicentre Randomised Controlled Trial. Lancet Oncol..

[54] Dirix P., Nuyts S., Van Den Bogaert W. (2006). Radiation-Induced Xerostomia in Patients with Head and Neck Cancer: A Literature Review. Cancer.

[55] Li Y., Taylor J.M.G., Ten Haken R.K., Eisbruch A. (2007). The Impact of Dose on Parotid Salivary Recovery in Head and Neck Cancer Patients Treated with Radiation Therapy. Int. J. Radiat. Oncol. Biol. Phys..

[56] Meyer S., Chibly A.M., Burd R., Limesand K.H. (2017). Insulin-Like Growth Factor-1-Mediated DNA Repair in Irradiated Salivary Glands Is Sirtuin-1 Dependent. J. Dent. Res..

[57] Mitchell G.C., Fillinger J.L., Sittadjody S., Avila J.L., Burd R., Limesand K.H. (2010). IGF1 Activates Cell Cycle Arrest Following Irradiation by Reducing Binding of ΔNp63 to the P21 Promoter. Cell Death Dis..

[58] Jensen S.B., Vissink A., Limesand K.H., Reyland M.E. (2019). Salivary Gland Hypofunction and Xerostomia in Head and Neck Radiation Patients. JNCI Monogr..

[59] Diwanji N., Bergmann A. (2018). An Unexpected Friend—ROS in Apoptosis-Induced Compensatory Proliferation: Implications for Regeneration and Cancer. Semin. Cell Dev. Biol..

[60] Ambudkar I.S. (2016). Calcium Signalling in Salivary Gland Physiology and Dysfunction. J. Physiol..

[61] Vinke J., Kaper H.J., Vissink A., Sharma P.K. (2020). Dry Mouth: Saliva Substitutes Which Adsorb and Modify Existing Salivary Condition Films Improve Oral Lubrication. Clin. Oral Investig..

[62] Furness S., Worthington H.V., Bryan G., Birchenough S., McMillan R. (2011). Interventions for the Management of Dry Mouth: Topical Therapies. Cochrane Database Syst. Rev..

[63] Mouly S., Salom M., Tillet Y., Coudert A.C., Oberli F., Preshaw P.M., Desjonquères S., Bergmann J.F. (2007). Management of Xerostomia in Older Patients: A Randomised Controlled Trial Evaluating the Efficacy of a New Oral Lubricant Solution. Drugs Aging.

[64] Carpenter G., Bozorgi S., Vladescu S., Forte A.E., Myant C., Potineni R.V., Reddyhoff T., Baier S.K. (2019). A Study of Saliva Lubrication Using a Compliant Oral Mimic. Food Hydrocoll..

[65] Saitoh E., Taniguchi M., Ochiai A., Kato T., Imai A., Isemura S. (2017). Bioactive Peptides Hidden in Human Salivary Proteins. J. Oral Biosci..

[66] Loo J.A., Yan W., Ramachandran P., Wong D.T. (2010). Comparative Human Salivary and Plasma Proteomes. J. Dent. Res..

[67] Wagner C.E., Wheeler K.M., Ribbeck K. (2018). Mucins and Their Role in Shaping the Functions of Mucus Barriers. Annu. Rev. Cell Dev. Biol..

[68] Ince D., Lucas T.M., Malaker S.A. (2022). Current Strategies for Characterization of Mucin-Domain Glycoproteins. Curr. Opin. Chem. Biol..

[69] Chawhuaveang D.D., Yu O.Y., Yin I.X., Lam W.Y.H., Mei M.L., Chu C.H. (2021). Acquired Salivary Pellicle and Oral Diseases: A Literature Review. J. Dent. Sci..

[70] Chaudhury N.M.A., Shirlaw P., Pramanik R., Carpenter G.H., Proctor G.B. (2015). Changes in Saliva Rheological Properties and Mucin Glycosylation in Dry Mouth. J. Dent. Res..

[71] Alliende C., Kwon Y.J., Brito M., Molina C., Aguilera S., Pérez P., Leyton L., Quest A.F.G., Mandel U., Veerman E. (2008). Reduced Sulfation of MUC5B Is Linked to Xerostomia in Patients with Sjögren Syndrome. Ann. Rheum. Dis..

[72] Winter C., Keimel R., Gugatschka M., Kolb D., Leitinger G., Roblegg E. (2021). Investigation of Changes in Saliva in Radiotherapy-Induced Head Neck Cancer Patients. Int. J. Environ. Res. Public Health.

[73] Ohyabu N., Kakiya K., Yokoi Y., Hinou H., Nishimura S.I. (2016). Convergent Solid-Phase Synthesis of Macromolecular MUC1 Models Truly Mimicking Serum Glycoprotein Biomarkers of Interstitial Lung Diseases. J. Am. Chem. Soc..

[74] Cherian R.M., Jin C., Liu J., Karlsson N.G., Holgersson J. (2016). Recombinant Mucin-Type Fusion Proteins with a Galα1,3Gal Substitution as Clostridium Difficile Toxin A Inhibitors. Infect. Immun..

[75] Becker T., Dziadek S., Wittrock S., Kunz H. (2006). Synthetic Glycopeptides from the Mucin Family as Potential Tools in Cancer Immunotherapy. Curr. Cancer Drug Targets.

[76] Kwan C.S., Cerullo A.R., Braunschweig A.B. (2020). Design and Synthesis of Mucin-Inspired Glycopolymers. ChemPlusChem.

[77] Schlatterer R., Marczynski M., Hermann B., Lieleg O., Balzer B.N. (2025). Unfolding of von Willebrand Factor Type D Like Domains Promotes Mucin Adhesion. Nano Lett..

[78] An J., Jin C., Dėdinaitė A., Holgersson J., Karlsson N.G., Claesson P.M. (2017). Influence of Glycosylation on Interfacial Properties of Recombinant Mucins: Adsorption, Surface Forces, and Friction. Langmuir.

[79] Pornpitchanarong C., Rojanarata T., Opanasopit P., Ngawhirunpat T., Bradley M., Patrojanasophon P. (2022). Maleimide-Functionalized Carboxymethyl Cellulose: A Novel Mucoadhesive Polymer for Transmucosal Drug Delivery. Carbohydr. Polym..

[80] Martin-Alarcon L., Govedarica A., Ewoldt R.H., Bryant S.L., Jay G.D., Schmidt T.A., Trifkovic M. (2024). Scale-Dependent Rheology of Synovial Fluid Lubricating Macromolecules. Small.

[81] Weiand E., Koenig P.H., Rodriguez-Ropero F., Roiter Y., Angioletti-Uberti S., Dini D., Ewen J.P. (2024). Boundary Lubrication Performance of Polyelectrolyte-Surfactant Complexes on Biomimetic Surfaces. Langmuir.

[82] Mystkowska J., Karalus W., Sidorenko J., Dąbrowski J.R., Kalska-Szostko B. (2016). Biotribological Properties of Dentures Lubricated with Artificial Saliva. J. Frict. Wear.

[83] Riquelme N., Laguna L., Tárrega A., Robert P., Arancibia C. (2021). Oral Behavior of Emulsified Systems with Different Particle Size and Thickening Agents under Simulated Conditions. Food Res. Int..

[84] Mystkowska J., Łysik D., Klekotka M. (2019). Effect of Saliva and Mucin-Based Saliva Substitutes on Fretting Processes of 316 Austenitic Stainless Steel. Metals.

[85] Mystkowska J., Lysik D., Germaniuk M., Niemirowicz-Laskowska K., Bucki R. The Influence of PH and Temperature on Stability of Artificial Saliva Based on Porcine Gastric Mucin. Proceedings of the 15th International Conference Mechatronic Systems and Materials, MSM 2020.

[86] Schweigel H., Wicht M., Schwendicke F. (2016). Salivary and Pellicle Proteome: A Datamining Analysis. Sci. Rep..

[87] Vukosavljevic D., Custodio W., Buzalaf M.A.R., Hara A.T., Siqueira W.L. (2014). Acquired Pellicle as a Modulator for Dental Erosion. Arch. Oral Biol..

[88] Wong R.S., Bennick A. (1980). The Primary Structure of a Salivary Calcium-Binding Proline-Rich Phosphoprotein (Protein C), a Possible Precursor of a Related Salivary Protein A. J. Biol. Chem..

[89] Zentner A., Heaney T.G. (1995). An In Vitro Investigation of the Role of High Molecular Weight Human Salivary Sulphated Glycoprotein in Periodontal Wound Healing. J. Periodontol..

[90] Busscher H.J., Rinastiti M., Siswomihardjo W., Van Der Mei H.C. (2010). Biofilm Formation on Dental Restorative and Implant Materials. J. Dent. Res..

[91] Mystkowska J. (2016). Biocorrosion of Dental Alloys Due to Desulfotomaculum Nigrificans Bacteria. Acta Bioeng. Biomech..

[92] Sundararaj D., Venkatachalapathy S., Tandon A., Pereira A. (2015). Critical Evaluation of Incidence and Prevalence of White Spot Lesions during Fixed Orthodontic Appliance Treatment: A Meta-Analysis. J. Int. Soc. Prev. Community Dent..

[93] Ambikathanaya U.K., Swamy K.N.R., Gujjari A.K., Tejaswi S., Shetty S., Ravi M.B. (2022). Effect of Acrylic Removable Partial Denture in Caries Prevalence among Diabetic and Non-Diabetic Patients. J. Pharm. Bioallied Sci..

[94] Martínez-Hernández M., Reyes-Grajeda J.P., Hannig M., Almaguer-Flores A. (2023). Salivary Pellicle Modulates Biofilm Formation on Titanium Surfaces. Clin. Oral. Investig..

[95] Hannig M. (1999). Ultrastructural Investigation of Pellicle Morphogenesis at Two Different Intraoral Sites during a 24-h Period. Clin. Oral Investig..

[96] Ma G., Tang Y., Zeng Q., Zheng J. (2019). On Adhesion Mechanism of Salivary Pellicle-PDMS Interface. Biosurf. Biotribol..

[97] Gibbins H.L., Yakubov G.E., Proctor G.B., Wilson S., Carpenter G.H. (2014). What Interactions Drive the Salivary Mucosal Pellicle Formation?. Colloids Surf. B Biointerfaces.

[98] Hannig M. (1997). Transmission Electron Microscopic Study of In Vivo Pellicle Formation on Dental Restorative Materials. Eur. J. Oral Sci..

[99] Juriaanse A.C., Booij M., Arends J., Ten Bosch J.J. (1981). The Adsorption in Vitro of Purified Salivary Proteins on Bovine Dental Enamel. Arch. Oral Biol..

[100] Fischer N.G., Aparicio C. (2021). The Salivary Pellicle on Dental Biomaterials. Colloids Surf. B Biointerfaces.

[101] Hirsh S.L., McKenzie D.R., Nosworthy N.J., Denman J.A., Sezerman O.U., Bilek M.M.M. (2013). The Vroman Effect: Competitive Protein Exchange with Dynamic Multilayer Protein Aggregates. Colloids Surf. B Biointerfaces.

[102] Roach P., Farrar D., Perry C.C. (2005). Interpretation of Protein Adsorption: Surface-Induced Conformational Changes. J. Am. Chem. Soc..

[103] Brash J.L., Horbett T.A., Latour R.A., Tengvall P. (2019). The Blood Compatibility Challenge. Part 2: Protein Adsorption Phenomena Governing Blood Reactivity. Acta Biomater..

[104] Yakubov G.E., Macakova L., Wilson S., Windust J.H.C., Stokes J.R. (2015). Aqueous Lubrication by Fractionated Salivary Proteins: Synergistic Interaction of Mucin Polymer Brush with Low Molecular Weight Macromolecules. Tribol. Int..

[105] Boyd H., Gonzalez-Martinez J.F., Welbourn R.J.L., Gutfreund P., Klechikov A., Robertsson C., Wickström C., Arnebrant T., Barker R., Sotres J. (2021). A Comparison between the Structures of Reconstituted Salivary Pellicles and Oral Mucin (MUC5B) Films. J. Colloid Interface Sci..

[106] Nagasawa D., Azuma T., Noguchi H., Uosaki K., Takai M. (2015). Role of Interfacial Water in Protein Adsorption onto Polymer Brushes as Studied by SFG Spectroscopy and QCM. J. Phys. Chem. C.

[107] Khutoryanskiy V.V. (2011). Advances in Mucoadhesion and Mucoadhesive Polymers. Macromol. Biosci..

[108] Alaei S., Omidian H. (2021). Mucoadhesion and Mechanical Assessment of Oral Films. Eur. J. Pharm. Sci..

[109] Hombach J., Bernkop-Schnürch A. (2010). Mucoadhesive Drug Delivery Systems. Handb. Exp. Pharmacol..

[110] Smart J.D. (2005). The Basics and Underlying Mechanisms of Mucoadhesion. Adv. Drug Deliv. Rev..

[111] Andrews G.P., Laverty T.P., Jones D.S. (2009). Mucoadhesive Polymeric Platforms for Controlled Drug Delivery. Eur. J. Pharm. Biopharm..

[112] Cook S.L., Bull S.P., Methven L., Parker J.K., Khutoryanskiy V.V. (2017). Mucoadhesion: A Food Perspective. Food Hydrocoll..

[113] Abruzzo A., Vitali B., Lombardi F., Guerrini L., Cinque B., Parolin C., Bigucci F., Cerchiara T., Arbizzani C., Gallucci M.C. (2020). Mucoadhesive Buccal Films for Local Delivery of Lactobacillus Brevis. Pharmaceutics.

[114] Giordani B., Abruzzo A., Musazzi U.M., Cilurzo F., Nicoletta F.P., Dalena F., Parolin C., Vitali B., Cerchiara T., Luppi B. (2019). Freeze-Dried Matrices Based on Polyanion Polymers for Chlorhexidine Local Release in the Buccal and Vaginal Cavities. J. Pharm. Sci..

[115] Abruzzo A., Nicoletta F.P., Dalena F., Cerchiara T., Luppi B., Bigucci F. (2017). Bilayered Buccal Films as Child-Appropriate Dosage Form for Systemic Administration of Propranolol. Int. J. Pharm..

[116] Szekalska M., Wróblewska M., Trofimiuk M., Basa A., Winnicka K. (2019). Alginate Oligosaccharides Affect Mechanical Properties and Antifungal Activity of Alginate Buccal Films with Posaconazole. Mar. Drugs.

[117] Kumria R., Al-Dhubiab B.E., Shah J., Nair A.B. (2018). Formulation and Evaluation of Chitosan-Based Buccal Bioadhesive Films of Zolmitriptan. J. Pharm. Innov..

[118] Kraisit P., Limmatvapirat S., Luangtana-Anan M., Sriamornsak P. (2018). Buccal Administration of Mucoadhesive Blend Films Saturated with Propranolol Loaded Nanoparticles. Asian J. Pharm. Sci..

[119] Fernandes F.P., Fortes A.C., Da Cruz Fonseca S.G., Breitkreutz J., Ferraz H.G. (2018). Manufacture and Characterization of Mucoadhesive Buccal Films Based on Pectin and Gellan Gum Containing Triamcinolone Acetonide. Int. J. Polym. Sci..

[120] Nair A.B., Al-Dhubiab B.E., Shah J., Vimal P., Attimarad M., Harsha S. (2018). Development and Evaluation of Palonosetron Loaded Mucoadhesive Buccal Films. J. Drug Deliv. Sci. Technol..

[121] dos Santos Garcia V.A., Borges J.G., Osiro D., Vanin F.M., de Carvalho R.A. (2020). Orally Disintegrating Films Based on Gelatin and Pregelatinized Starch: New Carriers of Active Compounds from Acerola. Food Hydrocoll..

[122] Ryu J.H., Choi J.S., Park E., Eom M.R., Jo S., Lee M.S., Kwon S.K., Lee H. (2020). Chitosan Oral Patches Inspired by Mussel Adhesion. J. Control. Release.

[123] Rossi S., Vigani B., Bonferoni M.C., Sandri G., Caramella C., Ferrari F. (2018). Rheological Analysis and Mucoadhesion: A 30 Year-Old and Still Active Combination. J. Pharm. Biomed. Anal..

[124] Peck C.C. (2016). Biomechanics of Occlusion—Implications for Oral Rehabilitation. J. Oral Rehabil..

[125] Johnson W.W. (1959). The History of Prosthetic Dentistry. J. Prosthet. Dent..

[126] Kydd W.L., Daly C.H. (1982). The Biologic and Mechanical Effects of Stress on Oral Mucosa. J. Prosthet. Dent..

[127] Maruo Y., Nishigawa G., Irie M., Oka M., Hara T., Suzuki K., Minagi S. (2010). Stress Distribution Prevents Ischaemia and Bone Resorption in Residual Ridge. Arch. Oral Biol..

[128] Imai Y., Sato T., Mori S., Okamoto M. (2002). A Histomorphometric Analysis on Bone Dynamics in Denture Supporting Tissue under Continuous Pressure. J. Oral Rehabil..

[129] Mori S., Sato T., Hara T., Nakashima K., Minagi S. (1997). Effect of Continuous Pressure on Histopathological Changes in Denture-Supporting Tissues. J. Oral Rehabil..

[130] Chen J., Ahmad R., Li W., Swain M., Li Q. (2015). Biomechanics of Oral Mucosa. J. R. Soc. Interface.

[131] Kumakura S., Sakurai K., Tahara Y., Nakagawa K. (2011). Relationship between Buccal Mucosa Ridging and Viscoelastic Behaviour of Oral Mucosa. J. Oral Rehabil..

[132] Stokes I.A.F., Laible J.P., Gardner-Morse M.G., Costi J.J., Iatridis J.C. (2011). Refinement of Elastic, Poroelastic, and Osmotic Tissue Properties of Intervertebral Disks to Analyze Behavior in Compression. Ann. Biomed. Eng..

[133] Kindler S., Seebauer C., Mksoud M., Samietz S., Kocher T., Holtfreter B., Lucas C., Völzke H., Metelmann H.R., Rau A. (2023). Impact of Dental Restorations and Removable Prostheses on Potentially Malignant Oral Mucosal Disorders in the General Population. J. Prosthet. Dent..

[134] Abuhajar E., Ali K., Zulfiqar G., Al Ansari K., Raja H.Z., Bishti S., Anweigi L. (2023). Management of Chronic Atrophic Candidiasis (Denture Stomatitis)—A Narrative Review. Int. J. Environ. Res. Public Health.

[135] Jainkittivong A., Aneksuk V., Langlais R.P. (2010). Oral Mucosal Lesions in Denture Wearers. Gerodontology.

[136] Martori E., Ayuso-Montero R., Martinez-Gomis J., Viñas M., Peraire M. (2014). Risk Factors for Denture-Related Oral Mucosal Lesions in a Geriatric Population. J. Prosthet. Dent..

[137] Sun L., Liu J., Zhang L. (2020). Evaluation of Friction in Different Oral Restoration Materials and Its Influencing Factors. Mater. Express.

[138] Prinz J.F., de Wijk R.A., Huntjens L. (2007). Load Dependency of the Coefficient of Friction of Oral Mucosa. Food Hydrocoll..

[139] Suchatlampong C., Davies E., von Fraunhofer J.A. (1986). Frictional Characteristics of Resilient Lining Materials. Dent. Mater..

[140] Waters M.G.J., Jagger R.G., Polyzois G.L. (1999). Wettability of Silicone Rubber Maxillofacial Prosthetic Materials. J. Prosthet. Dent..

[141] Aspinall S.R., Parker J.K., Khutoryanskiy V.V. (2021). Oral Care Product Formulations, Properties and Challenges. Colloids Surf. B Biointerfaces.

[142] Zheng J., Zhou Z.R. (2007). Friction and Wear Behavior of Human Teeth under Various Wear Conditions. Tribol. Int..

[143] Zheng Y., Bashandeh K., Shakil A., Jha S., Polycarpou A.A. (2022). Review of Dental Tribology: Current Status and Challenges. Tribol. Int..

[144] Mystkowska J., Car H., Dabrowski J.R., Romanowska J., Klekotka M., Milewska A.J. (2018). Artificial Mucin-Based Saliva Preparations—Physicochemical and Tribological Properties. Oral Health Prev. Dent..

[145] Andrysewicz E., Mystkowska J., Kolmas J., Jałbrzykowski M., Olchowik R., Dabrowski J.R. (2012). Influence of Artificial Saliva Compositions on Tribological Characteristics of Ti-6Al-4V Implant Alloy. Acta Bioeng. Biomech..

[146] Mair L.H. (1992). Wear in Dentistry-Current Terminology. J. Dent..

[147] Lambrechts P., Debels E., Van Landuyt K., Peumans M., Van Meerbeek B. (2006). How to Simulate Wear? Overview of Existing Methods. Dent. Mater..

[148] Hussein M.A., Mohammed A.S., Al-Aqeeli N. (2015). Wear Characteristics of Metallic Biomaterials: A Review. Materials.

[149] Walczak M., Drozd K. (2016). Tribological Characteristics of Dental Metal Biomaterials. Curr. Issues Pharm. Med. Sci..

[150] Saha S., Roy S. (2023). Metallic Dental Implants Wear Mechanisms, Materials, and Manufacturing Processes: A Literature Review. Materials.

[151] Souza J.C.M., Henriques M., Teughels W., Ponthiaux P., Celis J.P., Rocha L.A. (2015). Wear and Corrosion Interactions on Titanium in Oral Environment: Literature Review. J. Bio-Tribo-Corros..

[152] Mystkowska J., Niemirowicz-Laskowska K., Łysik D., Tokajuk G., Dąbrowski J.R., Bucki R. (2018). The Role of Oral Cavity Biofilm on Metallic Biomaterial Surface Destruction–Corrosion and Friction Aspects. Int. J. Mol. Sci..

[153] Briscoe W.H. (2017). Aqueous Boundary Lubrication: Molecular Mechanisms, Design Strategy, and Terra Incognita. Curr. Opin. Colloid Interface Sci..

[154] Briscoe W.H., Titmuss S., Tiberg F., Thomas R.K., McGillivray D.J., Klein J. (2006). Boundary Lubrication under Water. Nature.

[155] Sarkar A., Andablo-Reyes E., Bryant M., Dowson D., Neville A. (2019). Lubrication of Soft Oral Surfaces. Curr. Opin. Colloid Interface Sci..

[156] Yu S., Zhong M., Xu W. (2023). In Vitro Oral Simulation Based on Soft Contact: The Importance of Viscoelastic Response of the Upper Jaw Substitutes. J. Texture Stud..

[157] Wang X., Chen J., Wang X. (2022). In Situ Oral Lubrication and Smoothness Sensory Perception Influenced by Tongue Surface Roughness. J. Sci. Food Agric..

[158] Reeh E.S., Aguirre A., Sakaguchi R.L., Rudney J.D., Levine M.J., Douglas W.H. (1990). Hard Tissue Lubrication by Salivary Fluids. Clin. Mater..

[159] Yadav R., Meena A. (2022). Comparative Investigation of Tribological Behavior of Hybrid Dental Restorative Composite Materials. Ceram. Int..

[160] Pailler-Mattei C., Vargiolu R., Tupin S., Zahouani H. (2015). Ex Vivo Approach to Studying Bio-Adhesive and Tribological Properties of Artificial Salivas for Oral Dryness (Xerostomia). Wear.

[161] Zembyla M., Liamas E., Andablo-Reyes E., Gu K., Krop E.M., Kew B., Sarkar A. (2021). Surface Adsorption and Lubrication Properties of Plant and Dairy Proteins: A Comparative Study. Food Hydrocoll..

[162] Kullaa-Mikkonen A., Sorvari T.E. (1985). A Scanning Electron Microscopic Study of the Dorsal Surface of the Human Tongue. Cells Tissues Organs.

[163] Cheng S., Gandevia S.C., Green M., Sinkus R., Bilston L.E. (2011). Viscoelastic Properties of the Tongue and Soft Palate Using MR Elastography. J. Biomech..

[164] Alsanei W.A., Chen J. (2014). Studies of the Oral Capabilities in Relation to Bolus Manipulations and the Ease of Initiating Bolus Flow. J. Texture Stud..

[165] Ranc H., Elkhyat A., Servais C., Mac-Mary S., Launay B., Humbert P. (2006). Friction Coefficient and Wettability of Oral Mucosal Tissue: Changes Induced by a Salivary Layer. Colloids Surf. A Physicochem. Eng. Asp..

[166] Harvey N.M., Yakubov G.E., Stokes J.R., Klein J. (2012). Lubrication and Load-Bearing Properties of Human Salivary Pellicles Adsorbed Ex Vivo on Molecularly Smooth Substrata. Biofouling.

[167] Hatton M.N., Levine M.J., Margarone J.E., Aguirre A. (1987). Lubrication and Viscosity Features of Human Saliva and Commercially Available Saliva Substitutes. J. Oral Maxillofac. Surg..

[168] Reeh E.S., Douglas W.H., Levine M.J. (1996). Lubrication of Saliva Substitutes at Enamel-to-Enamel Contacts in an Artificial Mouth. J. Prosthet. Dent..

[169] Bongaerts J.H.H., Rossetti D., Stokes J.R. (2007). The Lubricating Properties of Human Whole Saliva. Tribol. Lett..

[170] Yakubov G.E., McColl J., Bongaerts J.H.H., Ramsden J.J. (2009). Viscous Boundary Lubrication of Hydrophobic Surfaces by Mucin. Langmuir.

[171] Lee S., Müller M., Rezwan K., Spencer N.D. (2005). Porcine Gastric Mucin (PGM) at the Water/Poly(Dimethylsiloxane) (PDMS) Interface: Influence of PH and Ionic Strength on Its Conformation, Adsorption, and Aqueous Lubrication Properties. Langmuir.

[172] Douglas W.H., Reeh E.S., Ramasubbu N., Raj P.A., Bhandary K.K., Levine M.J. (1991). Statherin: A Major Boundary Lubricant of Human Saliva. Biochem. Biophys. Res. Commun..

[173] Harvey N.M., Carpenter G.H., Proctor G.B., Klein J. (2011). Normal and Frictional Interactions of Purified Human Statherin Adsorbed on Molecularly-Smooth Solid Substrata. Biofouling.

[174] Stokes J.R., Boehm M.W., Baier S.K. (2013). Oral Processing, Texture and Mouthfeel: From Rheology to Tribology and Beyond. Curr. Opin. Colloid Interface Sci..

